# Staying Alive: Individual Behavioral Variation Influences Survival, but Not Reproductive Success, in Female Group‐Living Ground Squirrels

**DOI:** 10.1002/ece3.71861

**Published:** 2025-07-28

**Authors:** Miyako H. Warrington, Annemarie van der Marel, Jennifer Sojka, Krista J. Shofstall, Jane M. Waterman

**Affiliations:** ^1^ School of Biological and Medical Sciences Oxford Brookes University Headington UK; ^2^ Departamento de Ecologıa Pontificia Universidad Católica de Chile Santiago Chile; ^3^ Institute of Ecology and Biodiversity (IEB) Santiago Chile; ^4^ Department of Biological Sciences University of Manitoba Winnipeg Manitoba Canada; ^5^ Mammal Research Institute, Department of Zoology and Entomology University of Pretoria Pretoria South Africa

**Keywords:** lifetime fitness, maternity, pace‐of‐life, personality, sciurid

## Abstract

Animals living in harsh or unpredictable environments adopt adaptive strategies to improve their fitness, with behavioral variation playing a key role in shaping individual outcomes. We examined whether between‐individual variation in behavioral traits (personality) was associated with reproductive success and survival in female Cape ground squirrels ( *Xerus inauris* ). Using a 10‐year dataset (2011–2021), we quantified behavioral expressions of the animal's response to trapping and handling (trap response, as a proxy for docility), trapping rate (trappability, for boldness) and the number of different trapping locations an animal was trapped at (trap diversity, for exploration) and examined their associations with (1) annual reproductive success, (2) lifetime reproductive success, (3) annual survival, and (4) on‐site persistence (a proxy for lifespan). Response measures taken during transfer from the cage, handling by a human observer, and whether individuals ran or walked after release were moderately repeatable. Trappability was also repeatable, while trap diversity was not. Trap response and trappability were positively correlated with survival, but not reproductive success. Females that easily transferred from the trap to the handling bag (more docile) had higher annual survival, while those that ran after release had longer lifespans. Individuals trapped at a higher rate (bolder) had higher annual survival. The absence of a relationship between behavioral traits and reproductive success in females suggests that other factors, such as group dynamics, social interactions, and maternal effects, may be more influential in explaining the high reproductive skew in female reproductive success. Overall, our findings highlight the role of individual behavioral variation in shaping survival outcomes while emphasizing the need for further research into the mechanisms driving reproductive success in this species.

## Graphical abstract

We investigated how individual behavioural traits—docility and boldness—affect survival and reproductive success in female Cape ground squirrels over a ten‐year period. While both traits influenced survival, with more docile and bolder individuals showing higher annual survival, neither trait predicted reproductive success. These findings suggest behavioral variation supports survival but that other factors likely drive reproductive outcomes in this species.

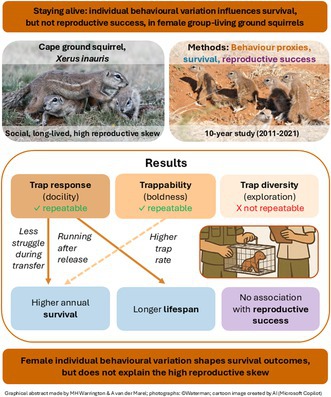

## 1 Introduction

Animals inhabiting harsh or unpredictable environments often evolve adaptive strategies that reflect their responses to varying ecological pressures. These adaptations include differences in life history traits and behaviors, particularly in reproductive and social strategies (Forcada et al. 2008; Fisher et al. 2021). For instance, such environments often select for a fast life history strategy, characterized by early maturation, high reproductive output, and shorter lifespans (Ricklefs and Wikelski 2002; Smith and Blumstein 2008; Dammhahn et al. 2018). These traits allow individuals to maximize their reproductive output within a limited time frame (Reid et al. 2003; Brumbach et al. 2009; Hampson et al. 2016; Rademaker et al. 2024), whereas individuals with a slow pace‐of‐life, characterized by delayed maturation, low fecundity, and longer lifespans, maximize their survival over a relatively longer timeframe (Ricklefs and Wikelski 2002; Smith and Blumstein 2008; Dammhahn et al. 2018). Yet, many species do not follow the pace‐of‐life syndrome (Bijleveld et al. 2014; Piquet et al. 2018; Laskowski et al. 2022; van der Marel et al. 2024); instead, they have a positive genetic correlation with longevity, fast growth, fast reproductive maturation, and higher quantities of offspring (Chang et al. 2024).

In addition to life‐history adaptations, some species employ social strategies to navigate ecological challenges. Group living, particularly communal breeding systems, enables individuals to reproduce and raise offspring collectively (Clutton‐Brock 2016; Ben Mocha et al. 2023). In such systems, cobreeders gain direct benefits derived from group living, such as enhanced predator protection, thermoregulatory benefits, and improved foraging efficiency (Lewis and Pusey 1997; Krause and Ruxton 2002), which can increase an individual's reproductive success and survival (Lewis and Pusey 1997). Despite these benefits, group living can also incur costs such as increased competition for food and mates, elevated aggression and conflict, and a greater risk of disease transmission (Krause and Ruxton 2002).

The costs and benefits of group living can be influenced and mediated via individual behaviors because behavioral traits shape how individuals engage with others (i.e., social structure), thereby influencing group dynamics (Whitehead 2008; Krause et al. 2010; Morrison et al. 2025). Behavioral traits can influence the acquisition of social roles or positions (Harten et al. 2018; Sasaki et al. 2018; Holtmann et al. 2021), which carry distinct costs and benefits (Silk 2007; Ellis et al. 2017). Consequently, group dynamics arise from individual behavioral tendencies that, in turn, influence individual survival and reproductive outcomes (Morrison et al. 2025).

The management of risk (survival) and reproduction depends not only on social factors, but also on environmental factors, such as predator density or resource stability (Dingemanse and Réale 2005). In unstable environments, either risk‐prone or exploratory individuals may benefit by finding novel or higher‐quality mating or feeding sites (Moran et al. 2017; Li et al. 2025). In contrast, risk‐averse individuals may do better in stable environments where sticking to familiar territory ensures consistent resource access and safety (Réale et al. 2007). Such trade‐offs underscore the adaptive flexibility of behavioral strategies, especially as they relate to reproduction and survival.

Many behavioral strategies are underpinned by consistent individual differences (Laskowski et al. 2022), collectively known as personality. These differences result in distinct behavioral phenotypes (Sih and Bell 2008). Traits such as exploration, boldness, aggressiveness, nurturing, and sociability can impact reproductive outcomes by guiding individuals toward strategies that best suit their individual characteristics and environmental contexts (Réale et al. 2010). Therefore, individual behavioral variation is a crucial factor shaping reproductive strategies and maximizing individual fitness.

Our objective was to study whether differences in consistent between‐individual behavioral variation are correlated to reproduction and survival in female Cape ground squirrels, *Xerus inauris* , using a 10‐year dataset. The Cape ground squirrel inhabits arid regions in southern Africa and is a highly social, diurnal, and semifossorial species (Waterman 1995). They have a distinctive social organization with permanent, year‐round matrilineal groups of one to six adult females and up to nine subadults of either sex. These female kin groups live separately from adult nonkin males (Waterman 1995; Pettitt and Waterman 2011). Social groups can also include up to three retained adult male offspring who delay dispersal (Waterman 1995; Scantlebury et al. 2008).

Group‐living in Cape ground squirrels offers several benefits: individuals receive allogrooming linked to parasite removal (Hillegass et al. 2010), sleep in shared burrow clusters that may provide thermoregulatory advantages (Waterman 1995; Herzig‐Straschil 1978; Gilbert et al. 2010; Scantlebury et al. 2012), and spend more time feeding due to collective vigilance (Unck et al. 2009). Cape ground squirrels show an uncommon life history among rodents as they live relatively long lives (Warrington et al. 2024) and have low reproductive output with high reproductive skew in both males (Manjerovic and Waterman 2015; Manjerovic et al. 2022; Warrington et al. 2022, 2024) and females (Herzig‐Straschil 1978; Waterman 1996; Hillegass et al. 2010). Females are sexually mature at 9–12 months, depending on the presence of other adult females or related adult males (Waterman 1996, 2002). Currently, it remains unknown what drives this high female reproductive skew; but perhaps behavioral variation may influence reproductive success, as it does in males of this species (Warrington et al. 2022, 2024). Identifying why some females reproduce more than others may reveal how this species, and similar ones, adapt to unpredictable and changing environments.

We hypothesized that behavioral traits, particularly docility, boldness, and exploration, may influence fitness in female Cape ground squirrels. Using trappability (as a proxy for boldness), trap diversity (for exploration), and response to trapping and handling (for docility), we assessed their associations with (1) annual reproductive success, (2) lifetime reproductive success, (3) annual survival, and (4) on‐site persistence (a proxy for lifespan). We predicted that females that struggled more during trapping and handling (less docile) or those trapped at a higher rate (higher trappability, bolder) would show higher reproductive success but lower survival because risk‐prone individuals may incur higher predation risk according to the life‐history trade‐offs associated with the pace‐of‐life syndrome hypothesis (Ricklefs and Wikelski 2002; Smith and Blumstein 2008; Dammhahn et al. 2018). Furthermore, if the number of traps an individual is trapped at (trap diversity) is linked to exploration, and if more exploratory females were able to acquire more resources, then we predicted that females with higher trap diversity would have higher survival and reproduction because both fitness proxies are influenced by resource acquisition (Haave‐Audet et al. 2021).

## 2 Methods

### 2.1 Study Site

Since 2002, trapping and ecological data have been collected on wild Cape ground squirrels at S.A. Lombard Nature Reserve (4600‐ha); located 18 km northwest of Bloemhof, South Africa (27° 36′ 6.48″S, 25° 28′ 0.48″ E), as part of an on‐going long‐term study. We used trapping data and specimen samples (for genetic kinship analysis) from May until August (austral winter) 2011–2021 to determine trappability, trap diversity, trapping responses, and fitness in individual female squirrels. However, the trap response was only assessed from 2014 to 2021.

At our study site, daily temperatures and rainfall fluctuate throughout the year. Mean temperature throughout the 24‐h period varies from approximately 2°C–29°C, with mean temperatures increasing by about 2°C over the last two decades (Scantlebury et al. 2012; Warrington and Waterman 2022). Rainfall is highly seasonal with pronounced wet and dry seasons, with precipitation confined mainly to the period from November to April (Van Zyl 1965). Seasonal rainfall amounts fluctuate highly from year to year, ranging from ca. 230–700 mm per year, with no trend changes over the course of the study (Warrington and Waterman 2022).

The study site includes two main habitat types, which vary in levels of predation and human activity (Unck et al. 2009). The human‐dominated area includes the field station and reserve building complex, and the natural areas are broadly divided into the natural floodplain habitat that is characterized by dry *Cymbopogon‐Themeda* veld and black soil turf veld, with patches of bush and pan areas (Van Zyl 1965). In years of high rainfall, vegetation and seeds, which are food sources for Cape ground squirrels, are abundant (O'Brien et al. 2021; Manjerovic et al. 2022). On‐site, natural predators of Cape ground squirrels include mammal, reptile, and avian predators, such as black‐backed jackals (C*anis mesomalas*), Cape cobra ( *Naja nivea* ), black‐shouldered kites ( *Elanus axillaris* ), and pale chanting goshawks ( *Melierax canorus* ).

### 2.2 Field Methods

From 2011 until 2021, we collected on‐site data on temperature and rainfall (mm) using thermometers and rain gauges, and trapped 434 different females over 2150 trapping occasions (Table 1). Throughout the field season, we conducted daily trapping rounds (2–4 rounds/day; 70 traps/round; 08:00–17:30) with Tomahawk live traps (15 × 15 × 50 cm, Tomahawk Live Trap Co., Tomahawk, WI, USA) baited with peanut butter and bird seed (Waterman 1995). Traps were fitted with shade covers to minimize heat stress and placed near burrow openings in burrow clusters, and checked routinely at approximately 2‐h intervals. Squirrels were marked with a PIT tag (AVID USA and Shenzhen XCC RFID Technology Co. Ltd. China) for permanent identification and a black hair dye mark (Rodol D; Lowenstein and Sons Inc., New York, NY, USA) for identification at a distance. For each trapped female we: (1) measured body mass to the nearest 5 g using a spring scale (Pesola AG, Baar, Switzerland); (2) measured spine length from the base of the skull to the base of the tail, with a tape measure; and (3) assessed sex and reproductive condition; adult females have developed nipples (which we checked for signs of lactation). We also palpated females to check for pregnancy. Additionally, we (4) scored an animal's reaction to trapping and handling by humans, following the methods of Réale et al. (2007). For subsequent parentage analysis, we collected 1–3 mm of skin from the tail tip of each new individual. We released each individual back into the area in which they were caught.

**TABLE 1:** The number of trap events where adult females were trapped, and the number of different females (unique tag identification) trapped from 2011 to 2021 at S.A. Lombard.

Year	Number of female trap events	Number of unique females (cumulative)
2011	114	49
2012	158	62
2013	378	72
2014	170	57
2015	248	75
2016	70	65
2017	72	61
2018	261	98
2019	400	119
2021	279	154
2011–21	2150	434

We calculated body condition, following Tranquillo et al. (2022), using principal component analysis (PCA), with both input variables (body mass, spine length) standardized (mean = 0, standard deviation = 1) prior to analysis. Body condition was defined as the second component (second component loadings: 0.707 for body mass, −0.707 for spine length) as heavier females had a higher score than lighter females of the same spine length.

### 2.3 Behavioral Proxies

We estimated trappability and trap diversity using trapping data from 2011 to 2019. Trappability is the probability that an individual is recaptured and measures the willingness to enter a baited trap. It is often used as a field measurement for boldness, as it may represent the risk an individual takes toward a novel object in their natural environment (Montiglio et al. 2012; Santicchia et al. 2021; Vanden Broecke et al. 2021). We measured trappability by calculating the encounter rate of each female per year, by dividing the total number of times a female was trapped by the total number of hours a trap was set in the vicinity of her burrow cluster and foraging range.

Trap diversity, the number of different burrow clusters visited, can be used as a proxy for exploration tendency (Santicchia et al. 2021), and gives insight into an animal's movement patterns (Vanden Broecke et al. 2021). We measured the total number of burrow clusters a female visited and standardized the measure by dividing the total clusters a female was trapped in divided by the total number of hours a trap was set in the vicinity of her burrow cluster and foraging range. Trap diversity likely represents the number of foraging sites a female used, as generally, females forage in the area near the burrow cluster where they are sleeping, with minimal overlap of feeding ranges with neighboring social groups (Waterman 1995).

We assessed the trap response on 1267 occasions for 361 unique females from 2014 to 2021. An individual's reaction to trapping and handling whereby individuals that are generally quiet, easy to manipulate, and do not struggle while being handled are generally considered to be docile (Réale et al. 2007). This response may also be associated with aggression, with more aggressive individuals struggling more during handling; but see Blumstein et al. (2013).

We assigned scores during four aspects of trapping and handling: approach, transfer, handling, and release. These behaviors were distinct and easy to qualify, and scores reflected the degree of reaction; high‐scoring individuals were less docile, and low‐scoring individuals were more docile. We scored docility as follows: (a) approach, the response of the subject during the handler's approach to the trap, as: 0—is quiet and still; 1—starts alarm calling and hissing when the handler approaches within 1 m of the trap; and 2—reacts to the handler from > 1 m, alarm calling, hissing, and thrashing; (b) transfer from the trap to the handling bag: 0—runs into the bag without protest; 1—resists, but enters the bag after 30 to 60 s, the handler may have to bang on the trap; 2—strongly resists bagging, the handler must reposition the trap or open the back and push the squirrel into the bag; (c) handling: 0—quiet and still, no perceptible reaction; 1—struggles, snorts, and alarm calls less than half the time, but handling is manageable; and 2—struggles, snorts, alarm calls more than half the time, making handling very difficult; and (d) upon release, the subject: 0—walks away; 1—runs away. After handling each squirrel, we released individuals into the area where they were captured.

### 2.4 Survival

We estimated survival by examining an individual's trapping history. Individuals were assumed to be dead if they were not retrapped in any subsequent years of the study. Because females do not disperse, they are either first trapped as juveniles or within a year of becoming an adult (if an individual was born shortly after the field season, they were caught as an adult in the subsequent field season). Most females (94%; 297/316) were resighted every year, and only 5% (15/316) of females after 1 year of being “missing” (un‐trapped), while 1% (3/316) and 0.3% (1/316) of females after a gap of 2 and 3 years, respectively. No females were missed for more than 4 years. Thus, females with 9 years of on‐site persistence would have been approximately 9 years old.

We quantified survival in two different ways: (1) annual survival, whether females survived until the following year, whereby a female that is never seen in all subsequent years in the trapping record is presumed dead; we attributed disappearances to death because females are philopatric, but we also note that we cannot determine the fate of all individuals that disappear, as is a common challenge in small mammal studies (Murray and Patterson 2006); (2) on‐site persistence, the number of years that a female was trapped on site as an adult.

### 2.5 Genetic Kinship Analysis

Maternity assessments followed those of Manjerovic and Waterman (2015). We extracted DNA from tail skin tissue collected during trapping using a DNeasy Kit (Qiagen Inc., Valencia, California). Individuals were genotyped using 19 species‐specific microsatellite loci (Shave and Waterman 2017) and we determined the maternity of genotyped juveniles using CERVUS v.3.0 (Marshall et al. 1998; Kalinowski et al. 2007), assuming an error rate of 90% in genotyping and that 95% of mothers were sampled. Combined nonexclusion probabilities were calculated separately for each year of analysis (based on the adult population in that year). We used parent pair analyses so that all individuals of possible breeding age (9 months, based on Pettitt and Waterman 2011; Waterman 2002) in the population were included in analyses. We ran 10,000 simulated bootstrap runs.

We confirmed assigned parents with manual checks of allelic matches to the offspring across all 19 loci. To determine the most parsimonious maternal assignment, each candidate was required to meet the following criteria, based on field observations and trapping records: (1) reproductive eligibility; candidates were aged in relation to the estimated gestation period of the offspring, and only individuals who were sexually mature at the time the offspring would have been in utero were retained as potential mothers; (2) group membership; mothers were required to be members of the same group as the offspring; (3) presence at birth; in cases where a candidate had no confirmed group affiliation, she was considered a potential mother only if trapping records confirmed she was alive during the offspring's birth period; and (4) spatial proximity; candidates must have been sighted within the same geographic area as the offspring. Only candidates meeting all four criteria were assigned as the biological mother.

## 3 Statistical Analyses

All statistical analyses were run in R v.4.4.0 (R Core Team 2025).

### 3.1 Repeatability Estimates

We examined repeatability using a Bayesian approach with Markov chain Monte Carlo multivariate GLMMs using the “MCMCglmm” package (Hadfield 2010), described by Dingemanse and Dochtermann (2013) and Houslay and Wilson (2017).

We ran a model to investigate within‐ and among‐individual variances and covariances for all four behavioral trap responses (approach, transfer, and handling fitted as Poisson, and release fitted as a categorical response). We included the following fixed factors that significantly affected the repeatability of docility behaviors in males (Warrington et al. 2022, 2024): (1) capture, whether the individual was being trapped for the first time (first time = 1, all captures thereafter = 0); (2) body condition; and (3) on‐site tenure, the number of years since the adult was first captured. As Cape ground squirrels have been trapped annually at this field site since 2002, females do not disperse (Waterman 1995) and the probability of “missing” a female if it is within the trapping area is low, on‐site tenure for all females is reasonably accurate and reflects their age. We also included (4) rainfall, the total precipitation from July of the previous year until June of the sampling year, which represents rainfall prior to the austral winter season and is associated with plant productivity (Van Zyl 1965). Body condition, tenure, and rainfall were *z*‐centered (mean = 0 and standard deviation = 1) before analysis. We also fitted three random effects: (a) tag, identity of the female squirrel; (b) handler ID, because the human handler may influence behavioral and physical measurements; and (c) area, where the individual was captured, as areas vary in the extent of predation pressure (Unck et al. 2009).

We calculated the adjusted repeatability of each behavioral response independently, by setting tag, area, handler ID, and the R‐matrix (residual covariance) to an “idh” structure, with approach, transfer, and handling modeled using a Poisson distribution (link = log), and release using a binomial distribution (link = logit), following Nakagawa and Schielzeth (2010) and Sanderson et al. (2015). We used noninformative inverse‐Wishart priors throughout and ran all models for 4,000,000 iterations, with a burn‐in of 5000 and thinning interval of 2000. Successive samples from the posterior distribution had low autocorrelation (most *r* < 0.01, while all were *r* < 0.05). To confirm model convergence, we visually inspected posterior distribution trace and density plots (Hadfield 2010) and calculated the autocorrelation between successive samples using the R package “coda” (Plummer et al. 2006). We then calculated the point estimates and credible intervals for each behavioral response from the posterior modes and highest posterior density (HPD) intervals. We considered behavioral traits to be repeatable if posterior distributions were symmetrical and lower HPD intervals were > 0.1.

Similarly, we fitted a model to investigate within‐ and among‐individual variances and covariances for trappability (encounter rate) and trap diversity (total burrow clusters visited), which were both log‐transformed first to be fitted as Gaussian responses. We included the following fixed factors: (1) body condition, (2) on‐site tenure, (3) rainfall, and the temperature variables of (4) maximum daily temperature and (5) minimum daily temperature, because temperature could affect animal activity and movement patterns (Moss and While 2021). All these fixed factors were *z*‐centered (mean = 0 and standard deviation = 1) prior to analysis. We also fitted the same three random effects (tag, handler ID, area) as repeatability models for docility. We then calculated the adjusted repeatability of each behavioral response independently as in the docility models, as described above.

### 3.2 Effects of Repeatable Behaviors on Reproductive Fitness

As the trap response during transfer, handling and release, trappability, and trap diversity were found to be repeatable, we fitted multivariate GLMMs using the R package “MCMCglmm” (Hadfield 2010) to investigate among‐individual variance and covariance for fitness and repeatable behaviors (Table 2). We ran separate model sets: one for trap response behaviors (M1–7), which were recorded each time an individual was trapped and handled (yielding multiple observations per individual per season) and another for trappability and trap diversity (M8–11), which were calculated once per individual per season.

**TABLE 2:** Multivariate GLMM models examining the effect of trappability (measured from 2011 to 2019) and trap response (measured from 2014 to 2021) on survival and fitness of females at S.A. Lombard Nature Reserve. The following variables were included in all models: Capture (trap response models only), maximum temperature, minimum temperature, rainfall, tenure, and body condition as fixed factors; tag, area, and handler ID as random factors. Note that trap diversity was not examined as it was found not to be repeatable.

Model	No. observations (N_unique females_)
**Trap response**	
M1—Annual Offspring: Continuous	914 (205)
M2—Annual Offspring: Binary	
M3—Lifetime Offspring: Continuous	707 (161)
M4—Lifetime Offspring: Binary	
M5—Annual Survival (Survival the following year)	899 (205)
M6—On‐site persistence: Continuous	698 (161)
M7—On‐site persistence: Binary	
**Trappability and trap diversity**	
M8—Annual Offspring: Continuous	513 (275)
M9—Annual Offspring: Binary	
M10—Total Offspring: Continuous	422 (229)
M11—Total Offspring: Binary	
M12—Annual Survival (Survival the following year)	513 (275)
M13—On‐site persistence: Continuous	422 (229)
M14—On‐site persistence: Binary	

For each model set, we ran separate models for each reproductive fitness measure: annual offspring continuous, the number of offspring produced that breeding season; annual offspring binary, whether an individual had any offspring that year, whereby zero offspring = 0, and ≥ one offspring = 1; total offspring continuous, the number of offspring produced during the individual's lifetime; and total offspring binary, whether any offspring were produced during the individual female's lifetime, whereby zero offspring = 0 and ≥ one offspring = 1. The binary variable of fitness was used to determine whether the explanatory fixed variables influenced whether a female bred at all either annually or over the course of their lifetime (to see if any of the fixed variables influence those individuals that never produce offspring). For total offspring analyses, we excluded individuals that were captured in 2021 (as they were still breeding), and all individuals that were first captured as adults in 2011 (as they may have produced offspring prior to the start of this study) as the inclusion of these individuals may lead to under‐estimating lifetime fitness. Thus, all females in these models have been tracked for their entire life from juvenile to adult.

For the trapping response models (M1‐7), transfer and handling were fitted as Poisson, and release was fitted as categorical. For the trappability and trap diversity models (M8‐14), trappability (encounter rate) was log‐transformed and fitted as Gaussian, and trap diversity (total burrow clusters) was fitted as Poisson. In all models, continuous fitness measures were fitted as Poisson, and binary fitness measures were fitted as categorical. We included the following fixed effects: capture number (for docility response variables only), tenure, rainfall, and body condition (z‐centered to improve model fit). Tag, area, and handler ID were fitted as random effects (Table 1).

For each model, we estimated within‐ and among‐individual covariance by fitting an unstructured “us” R‐matrix (within‐individual variation) for tag, and G‐matrix (among‐individual covariances). We used noninformative inverse Wishart priors and ran all models for 4,000,000 iterations, with a burn‐in of 5000 and a thinning interval of 2000. Successive samples from the posterior distribution had low autocorrelation (the majority were *r* < 0.02, while all were *r* < 0.05). We examined the correlations between response variables by standardizing model covariance response variables to a scale from −1 to 1 as described in Houslay and Wilson (2017); correlations were determined to be significant if the 95% credible interval of the correlation excluded zero.

### 3.3 Effects of Repeatable Behaviors on Survival

We examined among‐individual variance and covariance for survival and repeatable behaviors using the same methods as described above. We ran separate models for each measure of survival: (1) annual survival, presumed dead = 0, and survived = 1; (2) continuous on‐site persistence, the total number of years a female was trapped as an adult, and (3) binary on‐site persistence, whereby a female trapped for only 1 year = 0, and ≥ 2 years = 1. In both on‐site persistence models, we excluded all females that were captured in 2021 (i.e., still living at the end of the study, so we cannot use on‐site persistence as a proxy for lifespan).

Trap response, trappability, and trap diversity were fitted as described above, whereas on‐site persistence continuous was fitted as Poisson, and annual survival and on‐site persistence binary were fitted as categorical responses. We included the following fixed effects: capture number (docility models only), body condition, tenure (as a fixed effect for annual survival models, but for on‐site persistence models (lifespan), tenure was excluded because it is highly correlated to on‐site persistence). Rainfall was also included as a fixed effect; for persistence on‐site, annual rainfall prior to the field season was used (as in the fitness models above), and for annual survival, we used rainfall following the field season, as this variable represents the effect of rainfall on whether the female survived to the following field season. Tag, area, and handler ID were fitted as random effects (Table 1). Within‐ and among‐individual covariance estimates were determined using the method described above, with noninformative parametric‐expanded Wishart prior models run for 4,000,000 iterations, with a burn‐in of 5000 and thinning interval of 2000, and model covariance response variables standardized to a scale from −1 to 1.

## 4 Results

### 4.1 Repeatability Estimates

**FIGURE 1:**
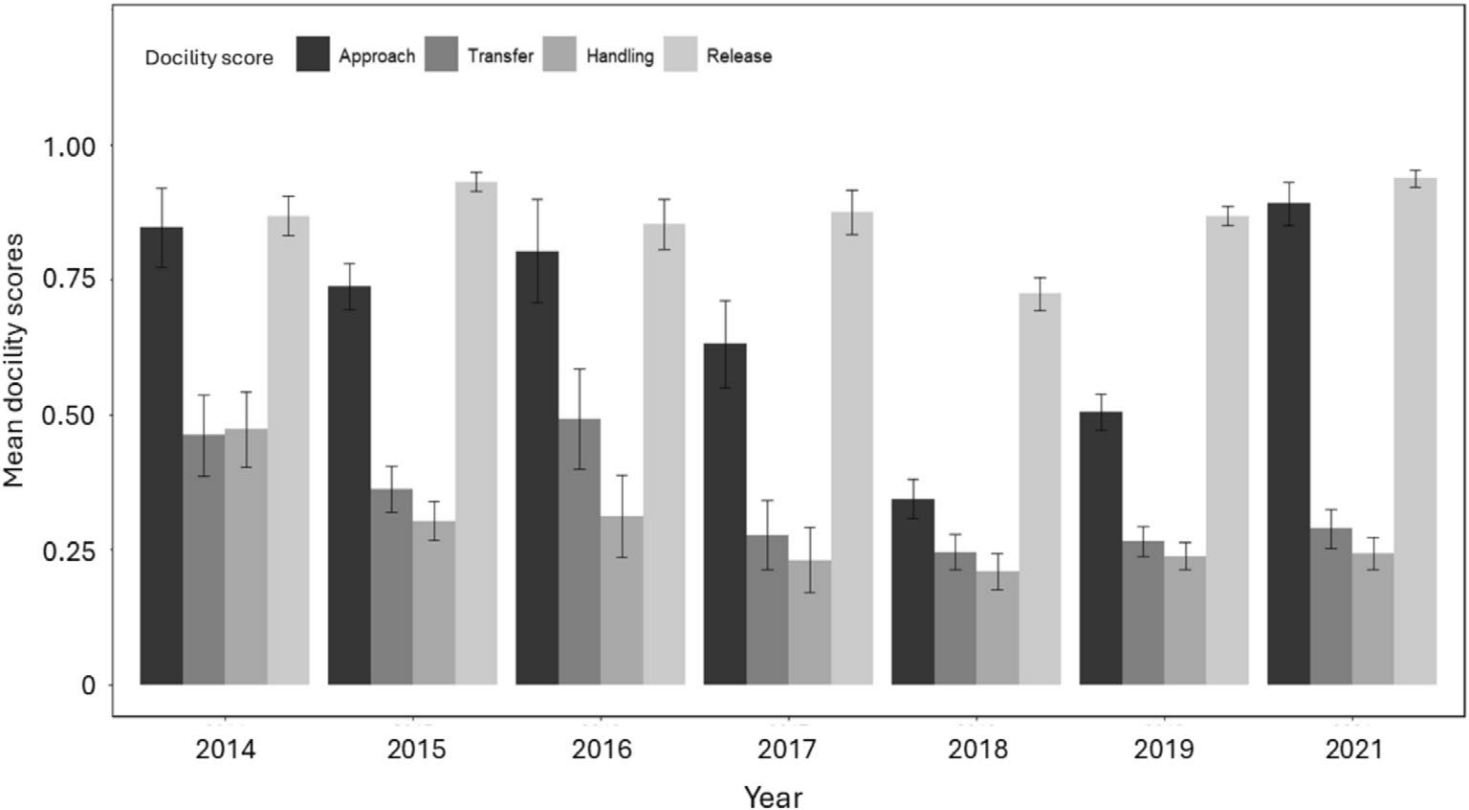
Mean ± SE trap response score during approach, transfer, handling, and release for all adult females sampled, by year. Individuals with low scores were considered more docile, and individuals with high scores were less docile.

From 2014 until 2021, during the austral winter, we scored the trap response 1267 times for 361 unique female Cape ground squirrels. On average, females were sampled 3.5 times each (range 1–24 times per individual). Average trap response, for all behaviors scored that year, varied from year to year (Figure 1, Table S2). We found moderate repeatability for transfer, handling, and release; we found no evidence of repeatability for approach (Table 3).

**TABLE 3:** Repeatability estimates for trap response, trappability, and trap diversity. We considered behavioral traits to be repeatable if posterior distributions were symmetrical and lower HPD intervals were > 0.1. Significant results are bolded.

Model	R	CI
Trap response—Approach	0.03	2.1 × 10^−7^–0.08
**Trap response—Transfer**	**0.46**	**0.35–0.53**
**Trap response—Handling**	**0.44**	**0.30–0.53**
**Trap response—Release**	**0.55**	**0.43–0.57**
**Trappability—Encounter rate**	**0.54**	**0.48–0.58**
Trap diversity	0.11	0.001–0.20

**FIGURE 2:**
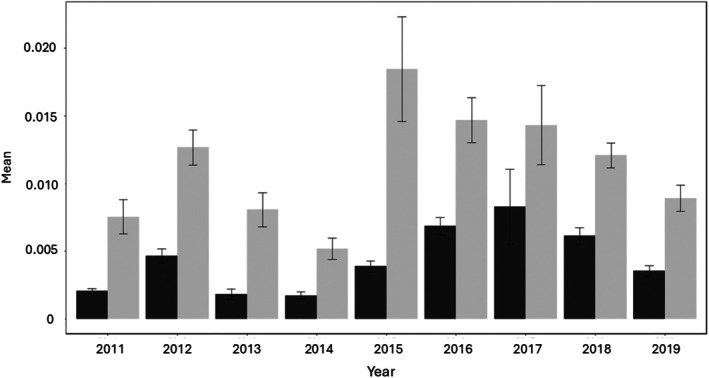
Mean ± SE trappability and trap diversity scores for all adult females sampled, by year. The black bar is the number of colonies per hour, and the gray bar is the number of trapping events per hour.

From 2011 until 2019, we calculated trappability and trap diversity (one calculation per female per year) for 300 unique females. On average, females were sampled over 1.93 years each (range 1–8 years per individual). Trappability and trap diversity, for all trapping events in each year, varied from year to year (Figure 2, Table S3). For the 139 unique females that were trapped for > 1 year (N_trap‐years_ = 377; total years trapped per female, range = 2–8 years, mean ± SE = 3.01 ± 0.11 years), trappability was repeatable, while trap diversity was not (Table 3).

### 4.2 Effects of Repeatable Behaviors on Reproductive Fitness

On an annual basis, most females (64%) had zero offspring, with few females having more than one offspring (22% of females had one offspring, and 9%, 3%, 1%, 0.02%, and 0.006% of females had 2, 3, 4, 5 and 6 offspring, respectively; Figure S1 and Table S4). Over their lifetime, only half (51%) of the females had offspring (Figure S2 and Table S5). Each female had on average ± SE, 1.44 ± 0.13 offspring over her lifetime.

Out of the 361 unique females (1267 observations) with a trap response score, we had fewer unique females where we determined each individual's tenure and body condition as well as their annual reproductive success (205 females, 914 observations) and lifetime reproductive success (161 females, 707 observations). We found no evidence of among‐individual covariance between any of the repeatable measures and annual fitness or lifetime fitness (Table 4).

**TABLE 4:** Among‐individual correlations and HPD lower and upper credible intervals, between repeatable behavioral traits (trap response during transfer, handling and release; trappability) and reproductive success and survival. Note that high trap response scores represent less docile females (see methods section). Significant correlations are bolded.

Model	Model number	Correlated traits	Among‐individual covariance	HPD lower CI	HPD upper CI
Annual offspring: continuous	M1	Transfer, annual offspring	−0.23	−0.48	0.01
	M1	Handling, annual offspring	−0.08	−0.39	0.20
	M1	Release, annual offspring	−0.03	−0.29	0.22
	M8	Trappability, annual offspring	0.23	−0.37	0.87
Annual offspring: binary	M2	Transfer, annual offspring binary	−0.24	−0.50	0.01
	M2	Handling, annual offspring binary	−0.01	−0.31	0.30
	M2	Release, annual offspring binary	−0.03	−0.29	0.22
	M9	Trappability, annual offspring binary	0.09	−0.79	0.93
Lifetime offspring: continuous	M3	Transfer, lifetime offspring	−0.20	−0.47	0.07
	M3	Handling, lifetime offspring	−0.18	−0.47	0.12
	M3	Release, lifetime offspring	0.27	−0.07	0.58
	M10	Trappability, lifetime offspring	0.32	−0.54	0.94
Lifetime offspring: binary	M4	Transfer, lifetime offspring binary	−0.14	−0.43	0.16
	M4	Handling, lifetime offspring binary	0.00	−0.34	0.31
	M4	Release, lifetime offspring binary	0.12	−0.24	0.44
	M11	Trappability, lifetime offspring binary	0.02	−0.85	0.90
Annual survival	M5	**Transfer, annual survival**	**−0.28**	**−0.55**	**−0.02**
	M5	Handling, annual survival	−0.11	−0.42	0.23
	M5	Release, annual survival	0.03	−0.27	0.32
	M12	Trappability, annual survival	0.36	−0.34	0.99
On‐site persistence: continuous	M6	Transfer, on‐site persistence	−0.16	−0.51	0.17
	M6	Handling, on‐site persistence	−0.13	−0.53	0.26
	M6	**Release, on‐site persistence**	**0.50**	**0.16**	**0.82**
	M13	Trappability, on‐site persistence	−0.04	−0.89	0.83
On‐site persistence: binary	M7	Transfer, on‐site persistence binary	−0.22	−0.51	0.11
	M7	Handling, on‐site persistence binary	−0.04	−0.38	0.31
	M7	Release, on‐site persistence binary	0.21	−0.17	0.54
	M14	**Trappability, on‐site persistence binary**	**0.57**	**0.28**	**0.84**

For trappability measures for only females that had been trapped > 1 year, and individuals where we had determined each their tenure and body condition, we had 275 females (513 observations) where we had determined their annual reproductive success and 229 females (422 observations) where we had determined their lifetime reproductive success. We found no evidence of among‐individual covariance between trappability and annual reproductive success, nor trappability and lifetime reproductive success (Table 4).

However, we found some evidence that maximum temperature, body condition, and total seasonal rainfall affected annual offspring (Table 5). In 3 out of 4 annual offspring models (M1, M8, M9), females had more offspring with warmer maximum (daytime) temperatures, and in 2 out of 4 models (M8, M9) had higher annual reproductive output with more rainfall (increased plant productivity). In two models (M1 and M2), females with higher reproductive output were in poorer body condition. However, we found no evidence that climatic variables affected lifetime reproductive success (Table 5).

**TABLE 5:** Estimated means (β) and 95% credible intervals (95% CI) for tenure, rainfall and body condition (fixed factors) on fitness and survival variables. Note that high trap response scores represent less docile females (see methods section). Effects of these fixed factors were also estimated for effects on transfer, handling and release, and trappability and are available Table S6. Significant results are bolded.

Model	Fixed effect	β	Lower 95% CI	Upper 95% CI	pMCMC
Trap response					
M1—Annual offspring: continuous	**Maximum temperature**	**0.43**	**0.18**	**0.71**	**0.002**
	Minimum temperature	−0.13	−0.34	0.06	0.21
	Total seasonal rainfall	−0.03	−0.16	0.08	0.58
	**Body condition**	**−0.36**	**−0.51**	**−0.23**	**< 0.001**
	Tenure	0.07	−0.08	0.24	0.38
M2—Annual offspring: binary	Maximum temperature	19.69	−2.25	46.44	0.07
	Minimum temperature	−5.36	−24.94	10.16	0.50
	Total seasonal rainfall	−2.12	−12.65	7.03	0.63
	**Body condition**	**−21.10**	**−36.06**	**−4.06**	**< 0.001**
	Tenure	4.50	−8.87	18.56	0.48
M3—Lifetime offspring: continuous	Maximum temperature	−0.16	−0.37	0.06	0.14
	Minimum temperature	−0.06	−0.17	0.07	0.33
	Total seasonal rainfall	0.04	−0.03	0.11	0.29
	Body condition	0.00	−0.06	0.08	0.91
	Tenure	0.11	−0.01	0.24	0.08
M4—Lifetime offspring: binary	Maximum temperature	−0.78	−1.70	0.10	0.13
	Minimum temperature	−0.37	−0.79	0.01	0.06
	Total seasonal rainfall	0.16	−0.29	0.54	0.67
	Body condition	0.41	−0.07	0.86	0.13
	Tenure	0.46	0.10	0.93	0.00
M5—Annual survival	Maximum temperature (S1)	−3.82	−10.57	1.76	0.19
	**Minimum temperature (S1)**	**12.34**	**−0.01**	**24.24**	**0.04**
	Total seasonal rainfall (S1)	−4.57	−11.98	1.00	0.12
	Body condition	−4.22	−10.40	0.52	0.09
	**Tenure**	**−63.23**	**−94.10**	**−21.76**	**< 0.001**
M6—On‐site persistence: continuous	Maximum temperature (S1)	−0.05	−0.11	0.02	0.17
	Minimum temperature (S1)	−0.02	−0.11	0.08	0.70
	Total seasonal rainfall (S1)	0.00	−0.08	0.07	0.92
	Body condition	−0.02	−0.07	0.04	0.48
M7—On‐site persistence: binary	Maximum temperature (S1)	−0.07	−0.45	0.18	0.80
	Minimum temperature (S1)	−0.22	−0.55	0.06	0.15
	Total seasonal rainfall (S1)	−0.09	−0.43	0.31	0.71
	Body condition	−0.07	−0.36	0.29	0.61
Trappability					
M8—Annual offspring: continuous	**Maximum temperature**	**0.29**	**0.11**	**0.49**	**0.001**
	Minimum temperature	0.09	−0.09	0.27	0.30
	**Total seasonal rainfall**	**0.22**	**0.04**	**0.38**	**0.01**
	Body condition	0.08	−0.22	0.07	0.30
	Tenure	−0.01	−0.17	0.12	0.90
M9—Annual offspring: binary	Maximum temperature	42.08	8.80	81.57	0.01
	Minimum temperature	16.98	−13.54	46.24	0.24
	Total seasonal rainfall	57.21	24.33	94.47	0.001
	Body condition	−3.57	29.20	25.01	0.79
	Tenure	0.89	−25.37	26.44	0.92
M10—Lifetime offspring: continuous	Maximum temperature	0.06	−0.15	0.28	0.58
	Minimum temperature	0.14	−0.04	0.33	0.16
	Total seasonal rainfall	0.12	−0.06	0.31	0.18
	Body condition	0.04	−0.11	0.21	0.58
	Tenure	0.14	−0.04	0.34	0.15
M11—Lifetime offspring: binary	Maximum temperature	0.02	−0.19	0.24	0.80
	Minimum temperature	0.08	−0.11	0.24	0.38
	Total seasonal rainfall	0.10	−0.08	0.31	0.33
	Body condition	0.06	−0.10	0.23	0.46
	Tenure	0.15	0.00	0.31	0.06
M12—Annual survival	Maximum temperature (S1)	9.02	−19.21	40.64	0.54
	Minimum temperature (S1)	13.15	−12.36	42.19	0.32
	Total seasonal rainfall (S1)	−21.35	−48.53	0.98	0.07
	Body condition	−12.66	−35.46	8.92	0.24
	Tenure	−28.74	−82.38	15.69	0.28
M13—On‐site persistence: continuous	Maximum temperature (S1)	−0.14	−0.27	0.01	0.08
	Minimum temperature (S1)	0.06	−0.07	0.18	0.36
	Total seasonal rainfall (S1)	0.00	−0.12	0.11	0.96
	Body condition	0.02	−0.09	0.12	0.78
M14—On‐site persistence: binary	Maximum temperature (S1)	−2.89	−13.65	7.61	0.57
	Minimum temperature (S1)	−1.41	−9.51	6.81	0.75
	Total seasonal rainfall (S1)	−0.84	−7.46	5.49	0.82
	Body condition	−0.32	−8.92	7.75	0.93

### 4.3 Effects of Repeatable Behaviors on Survival

Annual survival ranged from 0.43 to 0.75 of the sampled female population (Figure S3 and Table S7). On‐site persistence, a proxy for lifespan, ranged from 1 to 8 years. Most females (40%) survived only 1 year, with proportionally fewer females surviving subsequent years (on‐site persistence: 1 year—40% of females, 2 years—23%, 3 years—18%, 4 years—10%, 5 years—4%, 6 years—4%, 7 years—1%, 8 years—0.4%; Supporting Informations, Figure S4, Table S8).

We had 205 unique females (899 observations) where we had determined each individual's annual survival, and 161 females (698 observations) where we had determined on‐site persistence (lifespan), in addition to each individual's tenure and body condition. We found evidence of among‐individual covariance between transfer and annual survival (among‐individual covariance = −0.28, CI = −0.55 to −0.02; Table 4). Females that easily transferred from the cage to the handling bag (lower trap response scores/more docile) were more likely to have survived until the following year. We also found evidence of among‐individual covariance between release and on‐site persistence (lifespan). On average, females that ran after release had longer lifespans than those that walked away (among‐individual covariance = 0.50, CI = 0.16 to 0.82; Table 4). Trappability covaried with the binary measure of on‐site persistence (among‐individual covariance = 0.57, CI = 0.28 to 0.84; Table 4); females with higher encounter rates were more likely to have survived longer than their first year of adulthood.

We found some evidence that minimum temperature and tenure were associated with annual survival (Table 5). In the docility model (M5), females that experienced warmer minimum (night‐time) temperatures were more likely to have survived until the following year. Older females were more likely to have been unsighted the following year (presumed dead). Contrastingly, we found no evidence that climatic variables (maximum temperature, minimum temperature, total seasonal rainfall), body condition, or tenure (age) affected on‐site persistence (Table 5).

## 5 Discussion

We set out to study whether individual behavioral traits were correlated with reproductive success and survival in females of a communally breeding ground squirrel. We found no relationships between behavioral traits and annual or lifetime reproductive success, but some correlations with survival. Females that showed lower scores during transfer from the cage to the handling bag (more docile females) showed higher annual survival; females that ran instead of walked upon release from the trap (less docile females) showed longer lifespans, and females with higher trap encounter rates (bolder females) were more likely to have survived beyond their first year of adult life. Our results contrast with a study of male Cape ground squirrels in the same population, where more docile males had higher annual reproductive success, but docility was not correlated to survival (Warrington et al. 2024). Overall, our measured traits did not seem to explain the high reproductive skew and low reproductive output in the females of this species.

### 5.1 Individual Behavioral Variation and Reproductive Success

In contrast to our predictions, none of our measured behavioral traits were correlated with annual or lifetime reproductive success. Thus, it does not seem that these traits are under selection via reproductive success in Cape ground squirrel females. The relationship between individual behavioral traits and annual or lifetime reproductive success is also absent in other species (Smith and Blumstein 2008; Haave‐Audet et al. 2021; van der Marel et al. 2024).

Several mechanisms may explain the absence of this relationship in female Cape ground squirrels. First, the trade‐off between individual behavioral traits and reproductive success may be absent (Dochtermann 2023), and these traits may instead be important in a different context, such as group functioning, the communal care of offspring, or mate choice (Peignier et al. 2023). Second, resource availability may drive annual reproductive success more than individual behavioral variation (Clermont et al. 2023). For example, Siberian chipmunks, *Tamias sibiricus* , show an interaction between trappability and food availability, where bolder individuals show higher annual reproductive success with lower food availability and lower annual reproductive success with higher food availability (Le Cœur et al. 2015). Third, reproductive success may be influenced by mechanistic pathways instead of by one behavioral trait (Mutzel et al. 2013). Ultimately, the absence of the relationship between the measured behavioral traits and any reproductive measures in female Cape ground squirrels may reflect the complex interplay of ecological, social, and physiological factors, highlighting the need for further research to disentangle the specific drivers of reproductive success in this species.

Females had a high degree of reproductive skew and low annual reproductive output, which is consistent with earlier studies in this study population (Waterman 1996; Pettitt et al. 2008), and the males in this species (Warrington et al. 2024). Low reproductive success may be due to the unpredictable environment the Cape ground squirrels inhabit, which has been suggested to explain the high rate of reproductive failure (Waterman 1996). Particularly, in drought years, Cape ground squirrels do not reproduce (Waterman and Fenton 2000). Our results show some support that both maximum (daytime) temperature and cumulative amount of rainfall are important for annual reproductive success, as especially seen in arid environments, where a subsequent resource pulse follows rainfall (Previtali et al. 2009; Shenbrot 2014). However, our findings contrast with a study that compared reproductive success in this population of Cape ground squirrels and a population in a drier, hotter population in Namibia, which found that reproductive success did not differ despite the differences in rainfall between the two sites (Pettitt et al. 2008). Thus, our study highlights the importance of both between—and within‐study populations to elucidate the influence of climatic variables on reproductive success in a species.

Surprisingly, we found that females in poorer body condition showed higher reproductive success. This finding contrasts with studies where females in poor body condition showed lower reproductive success (Atkinson and Ramsay 1995; Wise and Jaeger 2021; Gajdošová et al. 2023). However, a lack of relationship between body condition and reproductive output could also have been influenced by the timing of sampling for the morphological features (spine length and body mass), which were used to assess body condition. Squirrels were measured following trapping, and a body condition was calculated for each trapping event. However, body condition prior to and following pregnancy cannot always be measured as females have spontaneous estruses and isolate themselves in natal burrows during birth and lactation (Waterman 1996), so perhaps the lower body condition associated with reproductive output reflects the cost of reproduction in females (Festa‐Bianchet 1998). Pregnancy seems costly for female Cape ground squirrels, given that ca. 70% of reproductive events (i.e., an individual female's estruses) do not result in successful return of offspring to the family burrow cluster (combined result of loss of pregnancy during gestation, birth or lactation; Waterman 1996).

Other potential constraints on reproductive success besides resources and climatic variables include predation, parasites, group features (e.g., group size and composition), and maternal effects. For example, in Cape ground squirrels, parasite removal resulted in greater reproductive success (Scantlebury et al. 2007; Hillegass et al. 2010). Furthermore, lower reproductive success has been observed in larger groups, possibly due to higher juvenile predation (Waterman 2002). Maternal effects, such as maternal age, weight, and experience, could also affect reproductive success (Gicquel et al. 2022; Roper et al. 2022; Yuen et al. 2024). Although in Cape ground squirrels, body mass has not been found to affect reproductive success, multiparous females have higher reproductive success (Pettitt et al. 2008), demonstrating a benefit to maternal experience. In summary, reproductive success in female Cape ground squirrels is more affected by factors other than individual behavioral variation.

### 5.2 Individual Behavioral Variation and Survival

We found some evidence that individual behavioral variation was correlated with annual survival and lifespan in female Cape ground squirrels. First, females that showed lower scores during transfer (more docile) had higher survival. Second, females that ran away upon release (less docile) showed a longer lifespan. Third, females trapped at a higher rate (more explorative) had higher annual survival.

We used trap response as a proxy for docility, but this response could be related to several other behavioral traits, including defensive boldness, social aggression (Blumstein et al. 2013), or risk‐taking behavior (Réale et al. 2009). Our findings suggest that more docile females (as measured by lower scores during handling) could have had survival benefits associated with risk aversion. Other studies have found that more docile animals also have higher survival (Réale et al. 2009), reinforcing the idea that more docile animals face less risk. Furthermore, risk‐averse females may have advantages in stable environments, where sticking to a proven territory and familiar resources ensures consistent self and offspring survival (Aronsson and Persson 2018). Additionally, Cape ground squirrels have many predators (Unck et al. 2009); thus, risk aversion can lead to strategies that prioritize survival over immediate reproductive efforts, which may be especially important because Cape ground squirrels have delayed reproduction (Waterman 2002). Cape ground squirrel females also have high rates of pregnancy loss, with more experienced (multiparous) females having higher reproductive success (Pettitt et al. 2008), and on average, females have fewer than two offspring over their lifetime (this study). This low reproductive success is particularly astounding because Cape ground squirrels can live up to 11 years (females = 8 years, this study; males = 11 years, Warrington et al. 2024). Altogether, these findings suggest that Cape ground squirrels may be employing a slow pace of life, as seen in large animals such as primates, cetaceans, and elephants (Dettmer and Chusyd 2023).

Higher docility has also been linked to the suite of traits associated with slower paces of life (Réale et al. 2009, 2010), including social living. Consequently, docility could be associated with social behaviors, such as social aggression (e.g., shy‐bold; Armitage and Van Vuren 2003), that can affect the acquisition of social benefits (Williams et al. 2023). As female Cape ground squirrels living in matrilineal kin groups benefit from mutual grooming resulting in reduced parasite loads (Hillegass et al. 2008), shared vigilance (Edwards and Waterman 2011), and communal care of young (Waterman 1995, 1996), being docile could allow females to obtain social benefits that can have downstream effects on fitness proxies. For example, in yellow‐bellied marmots ( *Marmota flaviventris* ), shyer females were more likely to live in territorial colonies and had higher lifetime fitness (Armitage and Van Vuren 2003). Thus, docility might be a behavioral trait that is advantageous in avoiding predation and receiving social benefits.

We also found that Cape ground squirrel females that entered a trap more often (higher trappability) had higher survival. If trappability was related to risk‐taking behavior (Réale et al. 2000, 2009), then our trappability results would contrast with what we found with docility. However, trappability is likely related to different ecological behavioral contexts than docility (assuming responses to human trapping and handling are related to ecological behaviors, but see Carter et al. (2013) and Brehm et al. (2020) for discussion). For example, trappability can be related to activity level, boldness, and acquisition of food resources (Boon et al. 2008; Santicchia et al. 2018), such that more trappable animals may have access to more food. In American red squirrels, *Tamiasciurus hudsonicus*, female squirrels that were trapped more often were more exploratory and had a greater number of food middens (Boon et al. 2008). Similarly, in Eurasian red squirrels, *Sciurus vulgaris* , survival was higher for bolder squirrels living in spruce forests as boldness appeared to confer an advantage to obtaining food in these food‐variable environments (Santicchia et al. 2018). Consequently, in Cape ground squirrels, the benefits of higher trappability may be related to food acquisition, especially as they receive a food reward of high‐calorie peanut butter mixed with seed.

In some species, increased trappability can be associated with not only the risks associated with entering a trap, but also the predation risk of being away from the safety of one's own territory (Boon et al. 2008). In our study, traps were placed close to burrow openings within burrow clusters. Trap diversity (the number of clusters an animal was trapped in) was not repeatable, which was not surprising because females were typically trapped within their burrow cluster as they show small foraging ranges and high site fidelity (Waterman 1995). Thus, high trappability is not likely associated with higher predation risk in our study, but instead, could be linked to movement behaviors related to resource acquisition or use (Hertel et al. 2020).

Females that ran away upon release showed a longer lifespan. Initially, we had interpreted running away upon release as less docile. However, in most trap events (86%, 1022/1182; Table S1), females ran after release. Running could be a response to the stress of handling (Réale et al. 2009), or it could be associated with an urgency to return to feeding (Cape ground squirrels spend approximately half their time feeding; Waterman 1995) or to move quickly toward an area of lower predation risk (e.g., close to their burrow; Unck et al. 2009). Given that both feeding rates and predation risk can affect survival (Prevedello et al. 2013), running following release can have impacts on lifespan independent of docile personalities.

Our study also highlights that other environmental factors can influence survival. Notably, in years with warmer nighttime (minimum) temperatures, annual survival was higher, suggesting that in this arid‐zone species, cold temperatures may compromise survival. Indeed, Cape ground squirrels experience significant thermoregulatory challenges due to cold nights and hot days, and their body temperatures fluctuate in response to these environmental extremes (Wilson et al. 2010; Scantlebury et al. 2012). They are slower to emerge from burrows and start diurnal activities on colder nights (Scantlebury et al. 2012), which suggests a thermoregulatory benefit to sociality via shared sleeping burrows (Waterman 1995), as seen in other burrowing and cosleeping mammals (Gilbert et al. 2010).

### 5.3 Sex‐Specific Associations

We found that docility was correlated to survival in females but not their reproductive success, in contrast with male Cape ground squirrels in this study population, where more docile and older males had higher reproductive success while docility had no association with survival (Warrington et al. 2024). Sex‐specific effects of behavioral variation have also been observed in other species; for example, in Bighorn sheep ( *Ovis canadensis* ), suggesting that males and females are shaped by different selective pressures (Réale et al. 2000, 2009; Hämäläinen et al. 2018). In Cape ground squirrels, these differences may be related to variation in sexual competition, as all females mate (Waterman 1996), while males face high levels of competition for females (operational sex ratio of 11 males to 1 female) (Waterman 1996, 1998; Manjerovic et al. 2022).

### 5.4 Further Work

The survival and fitness impacts of different individual behavioral traits can vary depending on proximal mechanisms, including internal (e.g., genetics and physiology) and external environmental drivers such as predation pressure (Réale and Festa‐Bianchet 2003), food availability (Dingemanse et al. 2004; Le Cœur et al. 2015; Santicchia et al. 2018), social dynamics (Both et al. 2005), and human disturbances (Brehm et al. 2019), or the interactions between multiple drivers (Wolf and Weissing 2010). Also, the relative impact of these factors may change over time, varying across different ages or life stages (Réale and Montiglio 2021). As a result, while certain traits may provide advantages in specific habitat conditions, the overall fitness of different behavioral phenotypes may balance out across a landscape with varying environmental pressures (Boon et al. 2007; Brehm et al. 2019) or timeframes. Cape ground squirrels consume a wide variety of food resources (Herzig‐Straschil 1978) and live in environments that vary in predation risk and human disturbance; thus, future studies examining behavioral variation should consider examining the distribution of food resources and predation risk across the landscape.

Climatic variables can also affect reproductive output or survival by directly altering individual behaviors, which in turn shape social interactions (e.g., agonistic or affiliative) and ultimately affect group size and structure (Fisher et al. 2021; Komdeur and Ma 2021). Consequently, changes in group size and structure can have impacts on reproduction and survival (Riehl and Smart 2022). However, the proximate mechanism linking climate‐induced behavioral changes to fitness consequences is complex (Moss and While 2021; Fisher et al. 2021). For example, behavioral changes related to high temperatures (i.e., heat stress) can affect grouping behavior via changes in movement rates associated with physiological stress, or by changing the accessibility and location of water sources and refugia (Moss and While 2021). Currently, we do not know how Cape ground squirrel social behavior changes with heat or water stress, and what temperature or humidity thresholds would lead to changes in behavioral patterns. Thus, further studies examining the relative contribution of plasticity versus consistency in individual behavioral traits (e.g., Montiglio et al. 2017), in relation to environmental factors, including interannual changes in climatic variables, would further our understanding of the role individual behavioral variation plays in maximizing fitness. Consequently, our study takes a first step in examining both between—and within year effects within a‐study population to elucidate the influence of climatic variables on reproductive success within a species.

Building on this understanding of how individual traits interact with environmental pressures, it is also important to consider how combinations of behaviors can further influence adaptive potential and fitness outcomes. Examining interactions or linkages between behavioral traits (i.e., behavioral syndromes, Sih et al. 2004) is a helpful further avenue of research because examining suites of linked behaviors can be both beneficial and limiting for an animal's fitness, because it affects how well an individual can adapt to different environmental challenges. For example, linked behaviors can allow for efficient decision‐making by streamlining responses to threats or opportunities, reducing hesitation and increasing survival chances (Sih et al. 2004). In contrast, linked behaviors can reduce flexibility, such that animals with rigid behavioral syndromes may struggle to adapt to changing environments (Sih and Bell 2008). Thus, understanding any behavioral linkages can elucidate how behavioral traits evolved and persist while providing insights into how species can thrive in dynamic ecosystems.

## 6 Conclusion

Our study highlights the complex links between individual behavioral traits, such as docility and boldness, and reproductive success and survival in female Cape ground squirrels. While these traits did not influence annual or lifetime reproductive success, they did affect survival, with less docile females exhibiting lower annual survival and bolder individuals showing longer lifespans. In contrast, reproductive success was more strongly associated with environmental factors such as temperature and rainfall, suggesting that external ecological conditions play a greater role in shaping reproductive outcomes than individual behavior. These findings contrast with previous research on males, where docility was linked to reproductive success, but not survival, indicating sex‐specific selective pressures in this species. Ultimately, our results emphasize the need for further research into the socioecological drivers of reproductive success and survival in group‐living and/or communally breeding species, particularly in unpredictable environments.

## Author Contributions

**Miyako H. Warrington:** conceptualization (equal), data curation (lead), formal analysis (lead), funding acquisition (supporting), investigation (supporting), methodology (equal), supervision (supporting), validation (lead), visualization (lead), writing – original draft (lead), writing – review and editing (lead). **Annemarie van der Marel:** conceptualization (supporting), methodology (supporting), writing – original draft (supporting), writing – review and editing (equal). **Jennifer Sojka:** conceptualization (equal), investigation (equal), methodology (equal), writing – review and editing (supporting). **Krista J. Shofstall:** investigation (equal), methodology (equal), writing – review and editing (supporting). **Jane M. Waterman:** conceptualization (equal), data curation (supporting), funding acquisition (lead), investigation (equal), methodology (supporting), project administration (lead), supervision (lead), writing – review and editing (supporting).

## Ethics Statement

All applicable international, national, and/or institutional guidelines for the care and use of animals were followed. All procedures were made in accordance with the American Mammal Association guidelines (Sikes and Gannon 2011; Sikes 2016) and were approved by the University of Manitoba's Animal Care and Use Committee (Permit #F14‐032, F18‐039). Northwest Parks and Tourism (South Africa) permitted the research.

## Conflicts of Interest

The authors declare no conflicts of interest.

## Supporting information


**Appendix S1:** ece371861‐sup‐0001‐AppendixS1.docx.

**Appendix S2:** ece371861‐sup‐0002‐AppendixS2.csv.

**Appendix S3:** ece371861‐sup‐0003‐AppendixS3.csv.

**Appendix S4:** ece371861‐sup‐0004‐AppendixS4.csv.

**Appendix S5:** ece371861‐sup‐0005‐AppendixS5.docx.

## Data Availability

All the required data are uploaded as Supporting Information.
