# Supplementary material for: Staying Alive: Individual Behavioral Variation Influences Survival, but Not Reproductive Success, in Female Group‐Living Ground Squirrels
Source: Ecol Evol. 2025 Jul 28;15(8):e71861. doi: 10.1002/ece3.71861 (PMC12304441; doi:10.1002/ece3.71861)
Supplement: Supplementary file 1 — Appendix S1: ece371861‐sup‐0001‐AppendixS1.docx. [file ECE3-15-e71861-s004.docx]

**Appendix**

**Capture history**

Table S1: Gaps in capture history of females (N=316) first and last captured from 2011-2018 at S.A.Lombard nature reserve. Trapping history is available until 2022, however, we excluded females last captured in 2019 (N=133), 2021 (N=76) and 2022 (N=102) as subsequent 3 years of history was unavailable, and because 2020 and 2021 had an unusual field schedule due to the COVID-19 pandemic travel restrictions created a gap in the trapping records (2020) and led to a shorter and later field season in 2021 at our study site.

|  | Skipped trapping | | | | | | |
| --- | --- | --- | --- | --- | --- | --- | --- |
|  | 0 years | | 1 year | 2 years | 3 years | 4+ years | Total females |
| Unique females | | 297 | 15 | 3 | 1 | 0 | 316 |
| Proportion | | 0.94 | 0.05 | 0.01 | 0.003 | 0 |  |

**Trapping response (docility)**

Table S2: Mean ± SE docility score during approach, transfer, handling and release for all adult females sampled, by year. Females were sampled 1267 times over the years, and this represented 361 unique females that were sampled on average 3.5 times each (range 1-24 times per individual).

|  |  |  | Mean ± SE trapping response score | | | |
| --- | --- | --- | --- | --- | --- | --- |
| Year | N sample size* | N unique females* | Approach | Transfer | Handling | Release |
| 2014 | 91 | 39 | 0.85±0.07 | 0.46±0.08 | 0.47±0.07 | 0.87±0.04 |
| 2015 | 221 | 79 | 0.74±0.04 | 0.36±0.04 | 0.30±0.04 | 0.93±0.02 |
| 2016 | 61 | 56 | 0.80±0.10 | 0.49±0.09 | 0.31±0.08 | 0.85±0.05 |
| 2017 | 65 | 58 | 0.63±0.08 | 0.28±0.06 | 0.23±0.06 | 0.88±0.04 |
| 2018 | 216 | 95 | 0.34±0.04 | 0.25±0.03 | 0.21±0.03 | 0.72±0.03 |
| 2019 | 374 | 123 | 0.51±0.03 | 0.26±0.03 | 0.24±0.02 | 0.87±0.02 |
| 2021 | 239 | 153 | 0.89±0.04 | 0.29±0.04 | 0.24±0.03 | 0.94±0.02 |
| 2014-2021 | 1267 | 361 |  |  |  |  |

*N sample size (N unique females) for approach in N_2018_ = 215(95); handling in N_2018_ = 209(95); for release N_2015_ = 211(78), N_2017_ = 59(57), N_2018_ = 204(95),N_2019_ = 363(123)

**Trappability and trap diversity**

Table S3: Mean ± SE trappability and trap diversity score by year. N sample size is the same as N unique females, as trappability and trap diversity is calculated for each female per year.

|  | |  | Mean ± SE score | |
| --- | --- | --- | --- | --- |
| Year | N sample size (unique females per year) | | Encounter/ hour | Colonies/ hour |
| 2011 | 45 | | 0.008±0.001 | 0.002±0.001 |
| 2012 | 48 | | 0.013±0.001 | 0.005±0.001 |
| 2013 | 62 | | 0.008±0.001 | 0.002±0.0004 |
| 2014 | 54 | | 0.005±0.001 | 0.002±0.0002 |
| 2015 | 64 | | 0.018±0.004 | 0.004±0.0004 |
| 2016 | 59 | | 0.015±0.002 | 0.007±0.0007 |
| 2017 | 60 | | 0.008±0.003 | 0.008±0.003 |
| 2018 | 84 | | 0.012±0.001 | 0.006±0.0006 |
| 2019 | 103 | | 0.009±0.001 | 0.004±0.0004 |
| 2011-2019 | | 300 |  |  |

**Reproductive success**

**
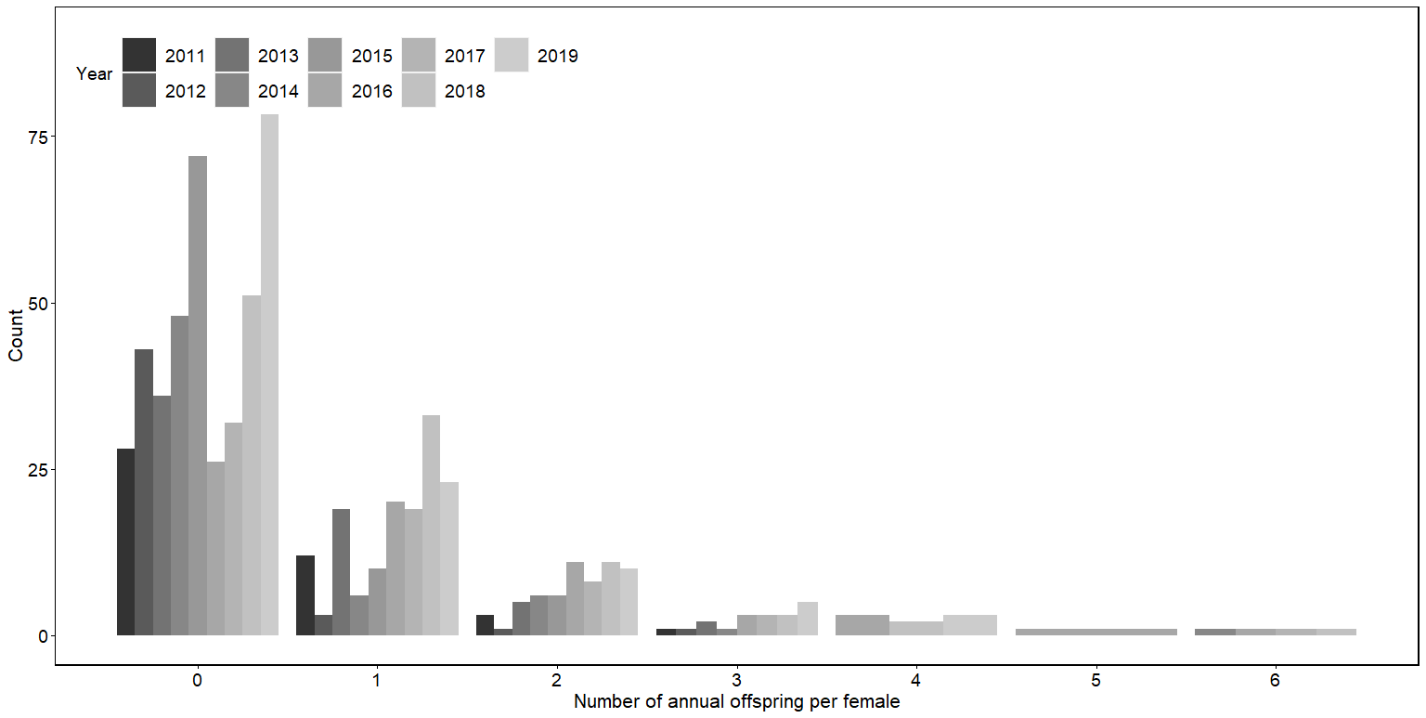
**

Fig.S1: Histogram of the number of annual offspring for each unique female by year (unique TagYear).

**
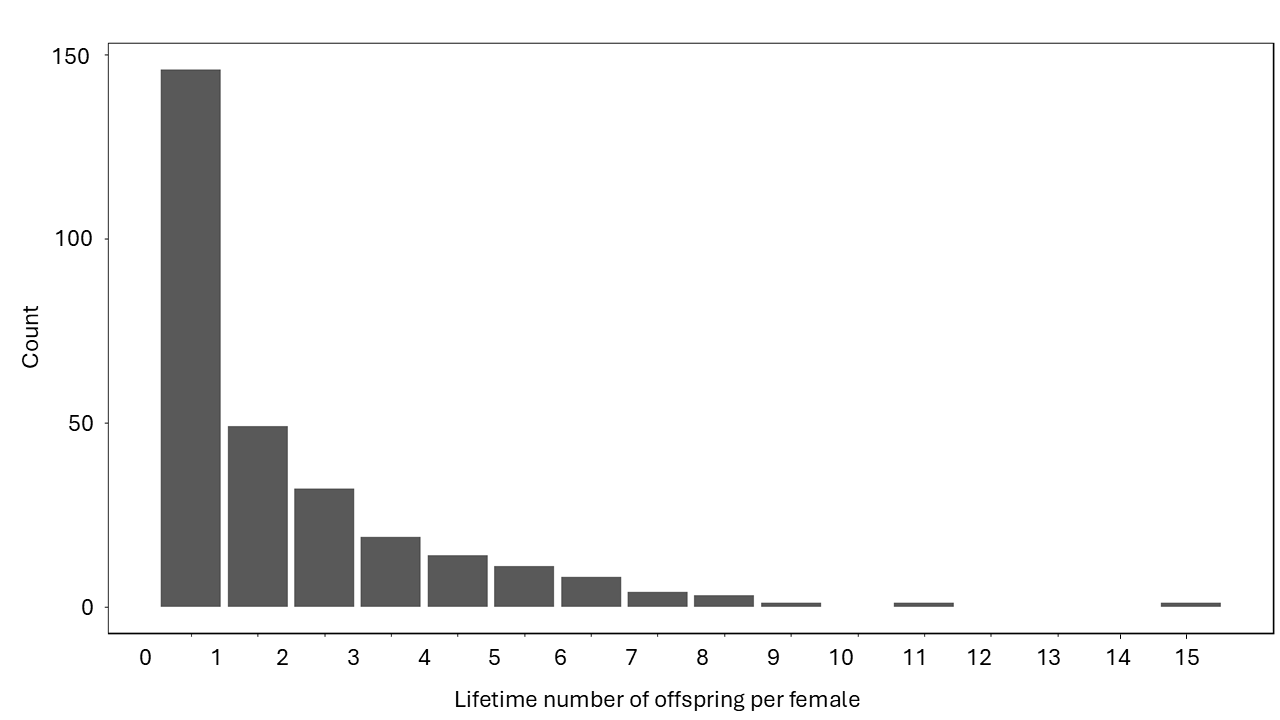
**

Fig.S2: Histogram of the total number of offspring over their lifetime for each unique female (unique Tag ID).

Table S4: Number of annual offspring for each unique female by year (unique TagID-Year).

|  | Number of females | | | | | |  |
| --- | --- | --- | --- | --- | --- | --- | --- |
| Year | Zero offspring | 1 offspring | 2 offspring | 3 offspring | 4 offspring | 5 offspring | 6 offspring |
| 2011 | 28 | 12 | 3 | 1 | 0 | 0 | 0 |
| 2012 | 43 | 3 | 1 | 1 | 0 | 0 | 0 |
| 2013 | 36 | 19 | 5 | 2 | 0 | 0 | 0 |
| 2014 | 48 | 6 | 6 | 1 | 0 | 0 | 1 |
| 2015 | 72 | 10 | 6 | 0 | 0 | 0 | 0 |
| 2016 | 26 | 20 | 11 | 3 | 3 | 1 | 1 |
| 2017 | 32 | 19 | 8 | 3 | 0 | 0 | 1 |
| 2018 | 51 | 33 | 11 | 3 | 2 | 0 | 1 |
| 2019 | 84 | 23 | 10 | 5 | 3 | 0 | 0 |
| **Total** | **420** | **145** | **61** | **19** | **8** | **1** | **4** |
| **Prop.** | **0.638** | **0.220** | **0.093** | **0.029** | **0.012** | **0.002** | **0.006** |

Table S5: Lifetime reproductive success (measured by the total number of offspring) for each unique female (unique Tag ID). For N = 57 females, we could not determine lifetime reproductive success because they were still alive at the end of the study (in 2021).

| Total # offspring | # unique females | |
| --- | --- | --- |
| 0 | 146 | |
| 1 | 49 | |
| 2 | 32 | |
| 3 | 19 | |
| 4 | 14 | |
| 5 | 11 | |
| 6 | 8 | |
| 7 | 4 | |
| 8 | 3 | |
| 9 | 1 | |
| 10 | 0 | |
| 11 | 1 | |
| 12 | 0 | |
| 13 | 0 | |
| 14 | 0 | |
| 15 | 1 | |
| Reproductive skew | 0.49 |  |

**Effect of fixed factors on fitness proxies**

Table S6: The effect of fixed factors on all behavioural response covariates (repeatable trapping response measures (approach, transfer, handling) and trappability (encounter rate). Significant effects are bolded.

| Model | Fixed effect | β | Lower 95% CI | Upper 95% CI | pMCMC |
| --- | --- | --- | --- | --- | --- |
| **Trapping response** |  |  |  |  |  |
|  |  |  |  |  |  |
| M1- Annual Offspring: Continuous | **Maximum temperature** | **0.43** | **0.18** | **0.71** | **0.002** |
|  | Minimum temperature | -0.13 | -0.34 | 0.06 | 0.21 |
|  | Total Seasonal Rainfall | -0.03 | -0.16 | 0.08 | 0.58 |
|  | **Body Condition** | **-0.36** | **-0.51** | **-0.23** | **<0.001** |
|  | Tenure | 0.07 | -0.08 | 0.24 | 0.38 |
| M2 - Annual Offspring: Binary | Maximum temperature | 19.69 | -2.25 | 46.44 | 0.07 |
|  | Minimum temperature | -5.36 | -24.94 | 10.16 | 0.50 |
|  | Total Seasonal Rainfall | -2.12 | -12.65 | 7.03 | 0.63 |
|  | **Body Condition** | **-21.10** | **-36.06** | **-4.06** | **<0.001** |
|  | Tenure | 4.50 | -8.87 | 18.56 | 0.48 |
| M3 - Lifetime Offspring: Continuous | Maximum temperature | -0.16 | -0.37 | 0.06 | 0.14 |
|  | Minimum temperature | -0.06 | -0.17 | 0.07 | 0.33 |
|  | Total Seasonal Rainfall | 0.04 | -0.03 | 0.11 | 0.29 |
|  | Body Condition | 0.00 | -0.06 | 0.08 | 0.91 |
|  | Tenure | 0.11 | -0.01 | 0.24 | 0.08 |
| M4 - Lifetime Offspring: Binary | Maximum temperature | -0.78 | -1.70 | 0.10 | 0.13 |
|  | Minimum temperature | -0.37 | -0.79 | 0.01 | 0.06 |
|  | Total Seasonal Rainfall | 0.16 | -0.29 | 0.54 | 0.67 |
|  | Body Condition | 0.41 | -0.07 | 0.86 | 0.13 |
|  | Tenure | 0.46 | 0.10 | 0.93 | 0.00 |
| M5 - Annual Survival | Maximum temperature (S1) | -3.82 | -10.57 | 1.76 | 0.19 |
|  | **Minimum temperature (S1)** | **12.34** | **-0.01** | **24.24** | **0.04** |
|  | Total Seasonal Rainfall (S1) | -4.57 | -11.98 | 1.00 | 0.12 |
|  | Body Condition | -4.22 | -10.40 | 0.52 | 0.09 |
|  | **Tenure** | **-63.23** | **-94.10** | **-21.76** | **<0.001** |
| M6 - On-site Persistence: Continuous | Maximum temperature (S1) | -0.05 | -0.11 | 0.02 | 0.17 |
|  | Minimum temperature (S1) | -0.02 | -0.11 | 0.08 | 0.70 |
|  | Total Seasonal Rainfall (S1) | 0.00 | -0.08 | 0.07 | 0.92 |
|  | Body Condition | -0.02 | -0.07 | 0.04 | 0.48 |
| M7 - On-site Persistence: Binary | Maximum temperature (S1) | -0.07 | -0.45 | 0.18 | 0.80 |
|  | Minimum temperature (S1) | -0.22 | -0.55 | 0.06 | 0.15 |
|  | Total Seasonal Rainfall (S1) | -0.09 | -0.43 | 0.31 | 0.71 |
|  | Body Condition | -0.07 | -0.36 | 0.29 | 0.61 |
| **Trappability** |  |  |  |  |  |
|  |  |  |  |  |  |
| M8- Annual Offspring: Continuous | **Maximum temperature** | **0.29** | **0.11** | **0.49** | **0.001** |
|  | Minimum temperature | 0.09 | -0.09 | 0.27 | 0.30 |
|  | **Total Seasonal Rainfall** | **0.22** | **0.04** | **0.38** | **0.01** |
|  | Body Condition | 0.08 | -0.22 | 0.07 | 0.30 |
|  | Tenure | -0.01 | -0.17 | 0.12 | 0.90 |
| M9 - Annual Offspring: Binary | **Maximum temperature** | **42.08** | **8.80** | **81.57** | **0.01** |
|  | Minimum temperature | 16.98 | -13.54 | 46.24 | 0.24 |
|  | **Total Seasonal Rainfall** | **57.21** | **24.33** | **94.47** | **0.001** |
|  | Body Condition | -3.57 | 29.20 | 25.01 | 0.79 |
|  | Tenure | 0.89 | -25.37 | 26.44 | 0.92 |
| M10 - Lifetime Offspring: Continuous | Maximum temperature | 0.06 | -0.15 | 0.28 | 0.58 |
|  | Minimum temperature | 0.14 | -0.04 | 0.33 | 0.16 |
|  | Total Seasonal Rainfall | 0.12 | -0.06 | 0.31 | 0.18 |
|  | Body Condition | 0.04 | -0.11 | 0.21 | 0.58 |
|  | Tenure | 0.14 | -0.04 | 0.34 | 0.15 |
| M11 - Lifetime Offspring: Binary | Maximum temperature | 0.02 | -0.19 | 0.24 | 0.80 |
|  | Minimum temperature | 0.08 | -0.11 | 0.24 | 0.38 |
|  | Total Seasonal Rainfall | 0.10 | -0.08 | 0.31 | 0.33 |
|  | Body Condition | 0.06 | -0.10 | 0.23 | 0.46 |
|  | Tenure | 0.15 | 0.00 | 0.31 | 0.06 |
| M12 - Annual Survival | Maximum temperature (S1) | 9.02 | -19.21 | 40.64 | 0.54 |
|  | Minimum temperature (S1) | 13.15 | -12.36 | 42.19 | 0.32 |
|  | Total Seasonal Rainfall (S1) | -21.35 | -48.53 | 0.98 | 0.07 |
|  | Body Condition | -12.66 | -35.46 | 8.92 | 0.24 |
|  | Tenure | -28.74 | -82.38 | 15.69 | 0.28 |
| M13 - On-site Persistence: Continuous | Maximum temperature (S1) | -0.14 | -0.27 | 0.01 | 0.08 |
|  | Minimum temperature (S1) | 0.06 | -0.07 | 0.18 | 0.36 |
|  | Total Seasonal Rainfall (S1) | 0.00 | -0.12 | 0.11 | 0.96 |
|  | Body Condition | 0.02 | -0.09 | 0.12 | 0.78 |
| M14 - On-site Persistence: Binary | Maximum temperature (S1) | -2.89 | -13.65 | 7.61 | 0.57 |
|  | Minimum temperature (S1) | -1.41 | -9.51 | 6.81 | 0.75 |
|  | Total Seasonal Rainfall (S1) | -0.84 | -7.46 | 5.49 | 0.82 |
|  | Body Condition | -0.32 | -8.92 | 7.75 | 0.93 |

**Survival**


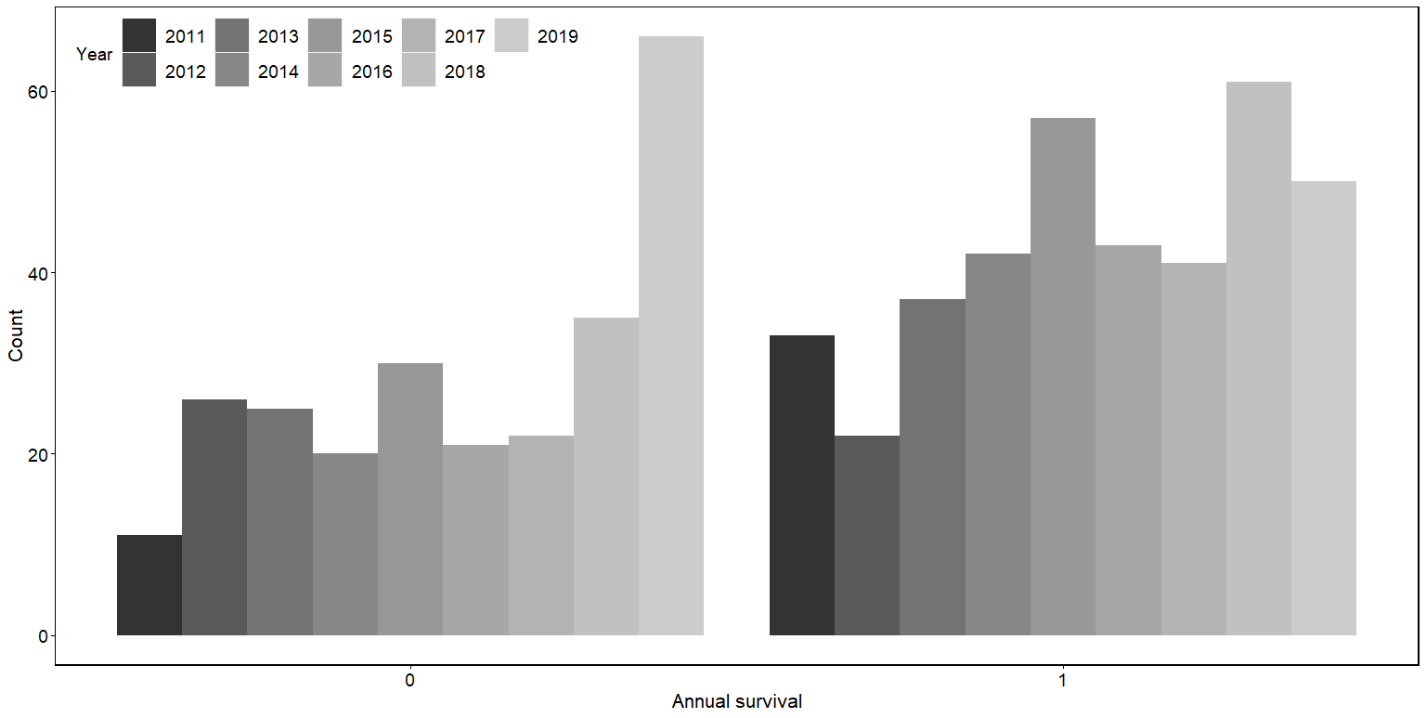


Fig. S3: Histogram of the annual survival (0 = disappeared/ not re-sighted; 1= trapped or observed) the followed year for each unique female per year (unique TagYear).


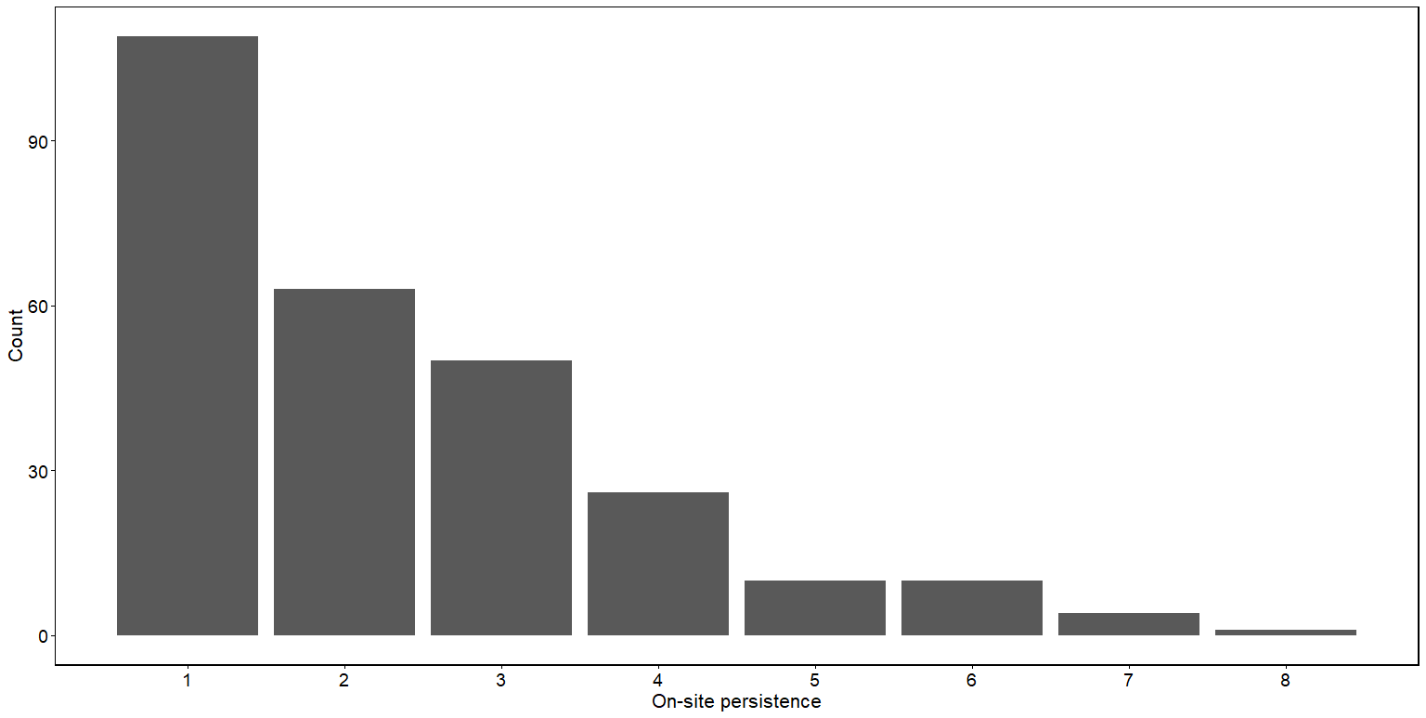


Fig. S4: On-site persistence, a proxy for lifespan for unique females (N=273) captured and scored for docility, trappability and/or trap diversity at S.A. Lombard Nature reserve from 2011-2021. We excluded all females (N=165) seen in 2021 (as they were alive at the end of the study). We accounted for age of first capture (adult versus juvenile/subadult).

Table S7: Annual survival: the number and proportion of females (unique TagYears) that were re-sighted (survived) the following year.

| Year | Number disappeared | Number survived | Prop survival |
| --- | --- | --- | --- |
| 2011 | 11 | 33 | 0.75 |
| 2012 | 26 | 22 | 0.46 |
| 2013 | 25 | 37 | 0.60 |
| 2014 | 20 | 42 | 0.68 |
| 2015 | 31 | 57 | 0.65 |
| 2016 | 21 | 43 | 0.67 |
| 2017 | 22 | 41 | 0.65 |
| 2018 | 35 | 61 | 0.64 |
| 2019 | 66 | 50 | 0.43 |

Table S8: On-site persistence, a proxy for lifespan for females (N=273) captured and scored for docility and/or trappability at S.A. Lombard nature reserve from 2011-2021.

| Persistence on-site | # Unique females | Prop of females |  |
| --- | --- | --- | --- |
| 1 | 109 | 0.40 | |
| 2 | 63 | 0.23 | |
| 3 | 50 | 0.18 | |
| 4 | 26 | 0.10 | |
| 5 | 10 | 0.04 | |
| 6 | 10 | 0.04 | |
| 7 | 4 | 0.01 | |
| 8 | 1 | 0.004 | |
| Total | 273 |  |  |
