# Supplementary material for: Staying Alive: Individual Behavioral Variation Influences Survival, but Not Reproductive Success, in Female Group‐Living Ground Squirrels
Source: Ecol Evol. 2025 Jul 28;15(8):e71861. doi: 10.1002/ece3.71861 (PMC12304441; doi:10.1002/ece3.71861)
Supplement: Supplementary file 5 — Appendix S5: ece371861‐sup‐0005‐AppendixS5.docx. [file ECE3-15-e71861-s002.docx]

**Metadata for the supplementary materials for**: ‘Staying alive: individual behavioural variation influences survival, but not reproductive success, in female group-living ground squirrels’

The supplementary materials include the following files:

1. **Supplementary_materials _metadata.docx** (this file): includes the description of the column names and description for the .csv files of the data used in the models presented in the manuscript:
2. **MODEL_FemaleDocilityFitness.csv**: the input file for the analyses of the effect of docility measures and other fixed factors on reproductive output in female Cape ground squirrels
3. **MODEL_FemaleDocilitySurvival.csv**: the input file for the analyses of the effect of docility measures and other fixed factors on survival in female Cape ground squirrels
4. **MODEL_FemaleTrappabilityREPRO&survival.csv**: the input file for the analyses of the effect of trappability and trap diversity measures and other fixed factors on reproductive output in female Cape ground squirrels

All model files were assembled in R using packages ‘dplyr’ and ‘tidyverse’ by Miya Warrington from the raw database files (trapping, morphological & docility measurement, parentage) of the Cape Ground Squirrel project lead by Jane M. Waterman. Rainfall data was obtained onsite by the S.A. Lombard Nature Reserve staff (Northwest Parks, South Africa).

Table S9: Description of column names in the model csv files

| Variable type | Column name | Description |
| --- | --- | --- |
| Random factor | Tag | Identity of the squirrel |
| Not used in models | Year | The year data was collected: initially examined as fixed or random factor in preliminary models |
| Random factor | Area | Area, the burrow cluster area where the individual was captured |
| Random factor | HandlerID | Human handler ID |
| Fixed factor | Approach | Docility score during approach to trap |
| Fixed factor | Transfer | Docility score during transferring animal from trap to handling bag |
| Fixed factor | Handling | Docility score during handling |
| Fixed factor | Release | Response of animal upon release: the subject 0 - walks away; 1 - runs away |
| Fixed factor | TotalColonies | Trap diversity: the total number of burrow clusters a female visited and standardized the measure by dividing the total clusters a female was trapped in divided by the total number of hours a trap was set in the vicinity of her burrow cluster and foraging range |
| Fixed factor | EncounterRate | Trappability: the encounter rate of each female per year; calculated by dividing the total number of times a female was trapped by the total number of hours a trap was set in the vicinity of her burrow cluster and foraging range |
| Fixed factor | CaptureNumber | Whether the individual was being trapped for the first time (first time = 1, all captures thereafter = 0) |
| Fixed factor | Tenure | The number of years since the adult was first captured |
| Fixed factor | TotalSeasonalPrecip | The total precipitation from July of the previous year until June of the sampling year |
| Fixed factor | S1YearTotalSeasRain | In survival models only: The total precipitation from July until June of the next year, representing the rainfall available during the year that the animal is to survive |
| Fixed factor | AvgBodyCond | Average body condtion for all measurements taken that season. Body condition was calculated as in Tranquillo et al. (2022), |
| Response variable | AnnualNumberOffspring | The number of offspring produced that breeding season |
| Response variable | AnnualNumberOffspringBinary | Whether an individual had any offspring that year, whereby zero offspring = 0, and ? one offspring = 1 |
| Response variable | LifetimeOffspring | Total offspring continuous: the number of offspring produced during the individual’s lifetime |
| Response variable | LifetimeOffspringBinary | Total offspring binary: whether any offspring were produced during the individual female’s lifetime, whereby zero offspring = 0 and ? one offspring = 1 |
| Response variable | AnnualSurvival | Whether females survived until the following year, whereby a female that is never seen in all subsequent years in the trapping record is presumed dead = 0, and females found in the trapping record are recorded as survived = 1 |
| Response variable | OnsitePersistance | On-site persistence continuous, the total number of years a female was trapped as an adult |
| Response variable | OnsitePersistenceBinary | On-site persistence binary, whether a female was trapped for > 1 year, whereby one year = 0, and ? two years = 1 |
